# Musashi2 promotes the progression of pancreatic cancer through a novel ISYNA1‐p21/ZEB‐1 pathway

**DOI:** 10.1111/jcmm.15676

**Published:** 2020-08-11

**Authors:** Lei Zhou, WeiWei Sheng, Chao Jia, Xiaoyang Shi, Rongxian Cao, Guosen Wang, Yiheng Lin, Fang Zhu, Qi Dong, Ming Dong

**Affiliations:** ^1^ Department of Gastrointestinal and Hernia and Abdominal Wall Surgery First Hospital of China Medical University Shenyang China; ^2^ Division of Cardiology The People’s Hospital of Liaoning Province Shenyang China; ^3^ Department of General Surgery The People’s Hospital of Liaoning Province Shenyang China

**Keywords:** cell biology, ISYNA1, MSI2, pancreatic cancer

## Abstract

Our previous studies found overexpression of Musashi2 (MSI2) conduced to the progression and chemoresistance of pancreatic cancer (PC) by negative regulation of Numb and wild type p53 (wtp53). Now, we further investigated the novel signalling involved with MSI2 in PC. We identified inositol‐3‐phosphate synthase 1 (ISYNA1) as a novel tumour suppressor regulated by MSI2. High MSI2 and low ISYNA1 expression were prevalently observed in 91 PC tissues. ISYNA1 expression was negatively correlated with MSI2 expression, T stage, vascular permeation and poor prognosis in PC patients. What's more, patients expressed high MSI2 and low ISYNA1 level had a significant worse prognosis. And in wtp53 Capan‐2 and SW1990 cells, ISYNA1 was downregulated by p53 silencing. ISYNA1 silencing promoted cell proliferation and cell cycle by inhibiting p21 and enhanced cell migration and invasion by upregulating ZEB‐1. However, MSI2 silencing upregulated ISYNA1 and p21 but downregulated ZEB‐1, which can be rescued by ISYNA1 silencing. Moreover, reduction of cell migration and invasion resulting from MSI2 silencing was significantly reversed by ISYNA1 silencing. In summary, MSI2 facilitates the development of PC through a novel ISYNA1‐p21/ZEB‐1 pathway, which provides new gene target therapy for PC.

## 1 INTRODUCTION

Pancreatic cancer (PC) is known as one of the most fatal tumours due to its aggressive malignant aggression in local invasion and distant metastasis. Nowadays, the 5‐year survival rate of PC remains 7%‐8% and the incidence rate remains increasing although surgery technique and radio‐chemotherapy are greatly improved.^1^, ^2^ It is imperative to find new molecular biomarkers which can predict the malignant biology and provide new therapeutic strategies for PC patients.

MSI2, a translational repressor, is of great importance for self‐renewal and pluripotency in embryonic stem cells via regulation of cloning efficiency and differentiation.^3^ Abnormal MSI2 expression has been found in chronic myeloid leukaemia, gastric cancer, hepatocellular cancer and colorectal cancers.^4^, ^5^, ^6^, ^7^ The findings in our former study indicated overexpression of MSI2 facilitated the progression and chemoresistance of PC through negative regulation of Numb and wild type p53 (wtp53).^8^, ^9^

Myoinositol, a kind of water‐soluble vitamin synthesized in cells, exhibits various functions, such as seawater acclimation, glucose‐lipid metabolism and tumour suppression.^10^, ^11^, ^12^, ^13^ Inositol‐3‐phosphate synthase 1 (ISYNA1) encodes a necessary limit‐enzyme in myoinositol biosynthesis and is a direct target of wtp53 in HCT116 and HepG2 cells.^15^ Meanwhile, ISYNA1 is closely related with the chemotherapeutic resistance in malignant melanoma.^16^ However, the coordinate role of ISYNA1 and MSI2 in the initiation of PC, to our knowledge, has not been reported, which would be investigated in current study.

## 2 MATERIALS AND METHODS

### 2.1 Tissue samples

Our study was ratified by the academic review committee from the first hospital of China Medical University. All patients signed sample informed consent and PC samples were collected after operation. We collected 91 paraffin‐embedded PC tissues and 57 paired adjacent pancreas in the First Hospital of China Medical University between March 2006 and October 2017. Meanwhile, we obtained additional 29 fresh paired PC and normal pancreatic tissues for mRNA and protein extraction. The normal adjacent pancreas was collected at more than two centimetres away from pancreatic cancer tissues. The research was conducted in accordance to the World Medical Association Declaration of Helsinki.

### 2.2 Cell lines and culture

We purchased Miapaca‐2, PANC‐1, AsPC‐1, BxPC‐2 and SW1990 cells from the Cell Bank of the Chinese Academy of Sciences (Shanghai, China), and Capan‐2 cell was received from the American Type Culture Collection. All PC cells were cultured with recommended 1640 or DMEM media including 10% foetal calf serum (HyClone), respectively. As described previously,^9^ SW1990 and Capan‐2 cells expressed wtp53 whereas Miapaca‐2, PANC‐1, AsPC‐1 and BxPC‐2 cells expressed mutant p53 (mtp53).

### 2.3 Immunohistochemistry

According to the methods described previously,^17^, ^18^ paraffin samples were used to perform immunohistochemistry (IHC). The intensity of staining was divided into 4 levels: 0 (negative), 1 (weak), 2 (medium), and 3 (strong), while extent of staining was divided into 5 levels: 0 (0%), 1 (1%‐25%), 2 (26%‐50%), 3 (51%‐75%) and 4 (76%‐100%) based on the percentage of the positively stained area, and three professional pathologists scored the final IHC staining. In addition, we only scored pancreatic ductal cells; pancreatic acinar and islet cells were excluded when scoring. The products of extent and intensity score were considered as final scores (0‐12). The final score > 4 was defined as high ISYNA1 and MSI2 expression.

### 2.4 Western blot

The whole protein lysates extracted by BCA method were boiling with loading buffer, added into 10% SDS‐polyacrylamide gels for electrophoresis, next proteins were transferred into polyvinylidene difluoride membranes(PVDM), then membranes were incubated by primary ISYNA1(Abcam), MSI2 (Abcam), Caspase‐3 (Abcam), p21(Abcam) and p53 (Proteintech), Bcl‐2 (Proteintech), Bax (Proteintech), ZEB‐1 (Proteintech), β‐Catenin (Proteintech), N‐cadherin (Proteintech), Snail‐1 (Proteintech), GAPDH (Proteintech) and β‐actin (Proteintech) antibodies at 4°C at least 6 hours or overnight. Membranes were immersed with secondary antibodies (Santa Cruz) at room temperature for 2 hours and finally detected by the ECL kit. The grey values of each band were analysed by Quantity One software. The experiments were repeated three times.

### 2.5 qRT‐PCR

1ml TRIzol reagent was added into each cell and tissue sample for extraction of total RNA under the manufacturer. Using nucleotide determination, RNA level of each sample was firstly maintained at the same level. Then total RNA was used to synthesize cDNA by the Expand Reverse Transcriptase Kit. Finally, ISYNA1 and GAPDH expression were detected by a Light Cycler 2.0 with the Light Cycler kit. The reaction conditions were denaturation at 95°C for 30 seconds, 45 cycles of amplification at 95°C for 5 seconds and dissolution at 60°C for 45 seconds. The primer sequences were seen in Table S1. The relative ISYNA1 expression was counted by ΔCT: ΔCT(ISYNA1)—ΔCT(GAPDH). The experiments were repeated three times.

### 2.6 Immunofluorescence staining assay

PC cells were seeded on coverslips overnight, then fixed by 10% paraformaldehyde, permeabilized with 0.1% Triton X‐100, blocked with 5% BSA, and hatched with ISYNA1 antibody (1:200; Abcam) at 4°C overnight. Then the coverslips were incubated with fluorescein isothiocyanate‐conjugated secondary antibody in darkness. Finally, the nuclear counterstain 4,6‐diamidino‐2‐phenylindole（DAPI）was added and the fluorescence pictures were picked by immunofluorescence microscopy. The experiments were repeated three times.

### 2.7 Construction of MSI2 silencing cell lines and ISYNA1 RNA interference

The sequence of Crispr‐cas9 and Crispr‐sgRNA were designed and synthesized by Genechem (Genechem Co, Ltd), while ISYNA1siRNA, p53siRNA and siRNA control were purchased from GenePharma (GenePharma Co, Ltd). Firstly, Capan‐2 and SW1990 cells were infected with Crispr‐cas9, next these cells were filtrated by puromycin (Sigma). Then the stable sublines were infected with MSI2‐sgRNA (sgMSI2‐1/sgMSI2‐2) and sgRNA control (scramble) to specifically silence target genes. Capan‐2, SW1990 and Miapaca‐2 cells were transiently transfected with ISYNA1siRNA, p53siRNA and siRNA control for 48‐72 hours with lipofectamine 3000 (Invitrogen) according to the manufacturer. The silencing efficiency of MSI2, ISYNA1 and p53 was verified by WB. All of target sequences were seen in Table S1.

### 2.8 Flow cytometric analysis of cell cycle

Total 5 × 10^6^ cells/mL in all pancreatic cancer cells were used for flow cytometry. 1 mL precooled 75% ethanol was added to fix the cells at 4°C overnight after transfection. Then cells were added with 500 μL premixed staining solution (500 μL buffer, 25 μL propidium iodide and 10 μL RNase A) and underwent water bath at 37°C for 30 minutes in darkness. CytoFLEX flow cytometry was applied to detect cell cycle with red fluorescence at wavelength of 488 nm and Flow Jo 7.6 software was used to analyse the results. Experiments were repeated three times.

### 2.9 Cell proliferation assays

After transfection with siRNA control and si‐ISYNA1‐1 for 48‐72 hours, the Capan‐2, SW1990 and Miapaca‐2 cells were inoculated to 96‐well plates at a suitable density (5000‐6000 cells per well) and cultured for 1‐4 days. Next, 20 μL MTT solution (5 mg/mL) was added into each well for 4 hours. Then removing cell supernatants and cells were treated with 100 μL dimethyl sulphoxide. The optical density (OD) value at 560 nm was finally calculated by the microplate reader. The experiments were repeated three times.

### 2.10 Cell migration and invasion assays

For cell migration assay, Capan‐2 and SW1990 cells were added to the top of chamber with growth media without FBS after transfection for 48‐72 hours or myoinositol (40 mg/mL) treatment for 48 hours. Growth media with 20% foetal bovine serum was added into the lower chamber as a chemoattractant. 24 hours later, the cells on the top of chamber were removed away Crystal Violet Hydrate (Sigma) under the instruction of manufacturer. And the cell invasion assay was conducted in an analogous method with the top of chamber pre‐coated with Matrigel (BD Biosciences). The images of cell migration and invasion were obtained via the microscope (Nikon Microphot‐FX) at 100× magnification; cells in every chamber were calculated under five random fields at 200× magnification. 5 × 10^6^ cells/mL and 2.5 × 10^6^ cells/mL of SW1990 cells and 2 × 10^6^ cells/mL and 1 × 10^6^ cells/mL of Capan‐2 cells were used to perform cell migration and invasion experiments, respectively. The experiments were repeated three times.

### 2.11 Statistical analysis

Statistical analyses were conducted by SPSS software 20.0 (SPSS). The differences between expression of MSI2 and ISYNA1 in pancreatic cancer and adjacent normal pancreatic tissues were analysed by *t* test. The clinicopathological significance of ISYNA1 and MSI2, and their relationship were estimated by chi‐squared and correlation analysis, respectively. Survival of patients was analysed by the Kaplan‐Meier curve, while Log‐rank test was used to evaluate the differences. The differences of cell proliferation and cycle, cell migration and invasion were analysed by *t* test. *P* < .05 was considered to be statistically significant.

## 3 RESULTS

### 3.1 Exceptional ISYNA1 expression in pancreatic cancer tissues and cells

**Figure 1:**
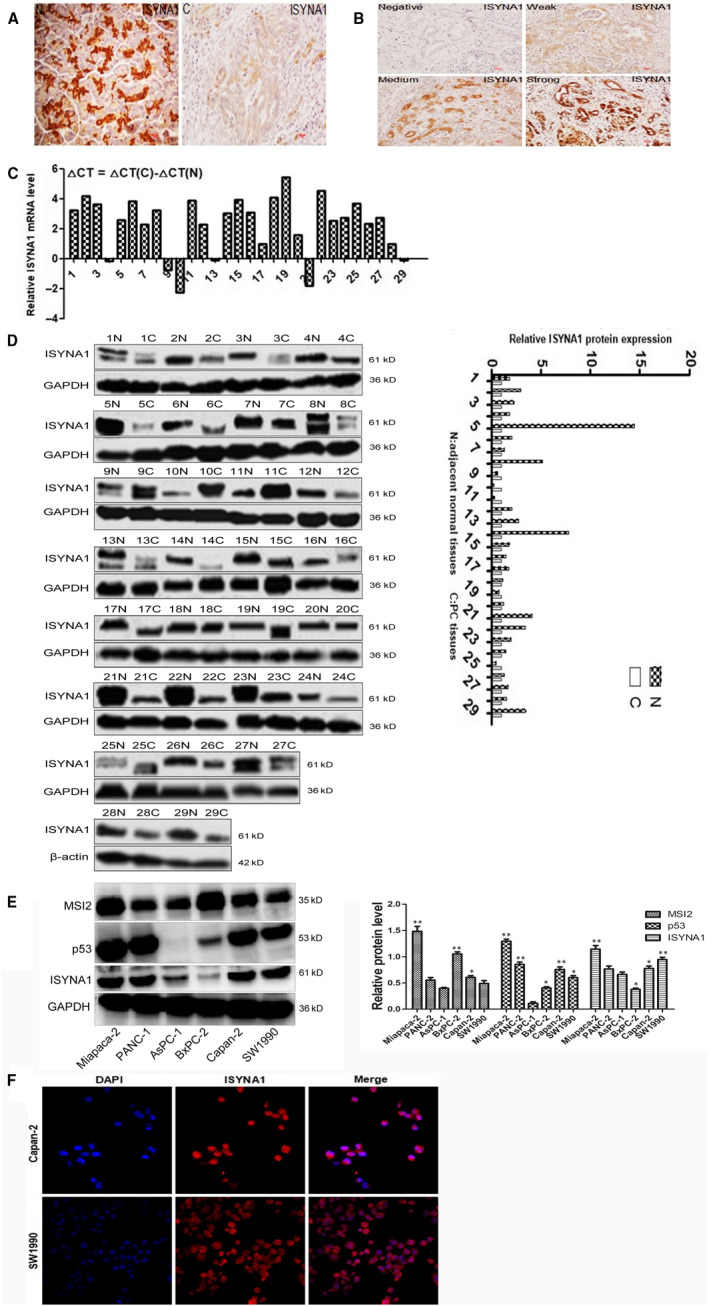
Expression of ISYNA1 in PC tissues and cell lines. A, Images of ISYNA1 expression in PC tissues (C) and adjacent normal tissues (N) by IHC (200×). B, Different ISYNA1 expression intensity in PC tissues (C) (200×). C and D, qRT‐PCR and WB analysis of ISYNA1 mRNA and protein in 29 cases of PC tissues (C) and paired normal tissues (N), respectively. E, MSI2, p53 and ISYNA1 protein expression in six PC cell lines as detected by WB. F, Localization of ISYNA1 protein in Capan‐2 and SW1990 by immunofluorescence staining. N: adjacent normal tissues; C: PC tissues. Bars indicate means ± SE, *P < .05, **P < .01

In 91 PC tissues and 57 adjacent normal pancreas, ISYNA1 was positively expressed in 36 PC tissues (36/91, 39.5%), which was much lower than that in 57 normal pancreas (33/57, 57.9%) (*P* < .01). Similarly, in 57 paired specimens, ISYNA1 expression in PC tissues (3.91 ± 2.38) was lower than that in paired normal tissues (5.72 ± 3.16) (*P* < .01) (Figure 1A,B). Meanwhile, both qRT‐PCR and WB showed ISYNA1 mRNA and protein level in 29 paired fresh pancreatic cancer tissues were lower than that in corresponding normal pancreatic tissues, respectively (*P* < .01; *P* = .001) (Figure 1C,D). In vitro, ISYNA1 protein was highly expressed in Miapaca‐2, Capan‐2 and SW1990 cells compared with other cell lines (Figure 1E). Immunofluorescence staining showed ISYNA1 was expressed in both nucleus and cytoplasm in pancreatic cancer cell lines (Figure 1F), which was consistent with the results in IHC assays.

### 3.2 Association between MSI2 and ISYNA1 expression with patients’ parameters and survival time

**Figure 2:**
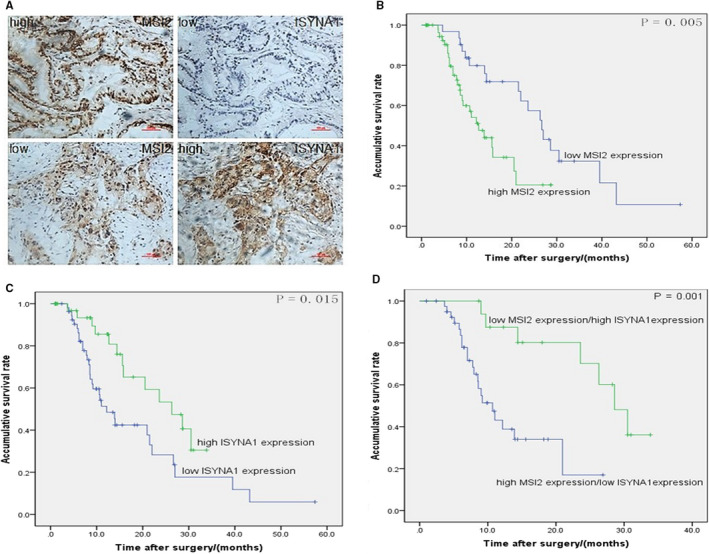
The relationship of MSI2 and ISYNA1 with the survival of 91 postoperative PC patients in IHC and Kaplan‐Meier analysis. A, IHC showed negative expression of MSI2 and ISYNA1 in PC tissues (200×). B and C, Kaplan–Meier survival curves in PC patients with high and low ISYNA1, MSI2 expression levels, respectively. D, The negative relationship of MSI2 and ISYNA1 was plotted against overall survival time

In our previous study, we proved that MSI2 overexpression was positively associated with tumour size, UICC stage and poor prognosis of pancreatic cancer patients.^8^ In current study, ISYNA1 expression was negatively related to T stage (*P* = .035) and vascular permeation (*P* = .030) in 91 PC patients (Table 1). Moreover, high expression of MSI2 with low ISYNA1 expression was found in serial pancreatic cancer sections and vice versa (Figure 2A). Correlation analysis identified a negative correlation between their expression in pancreatic cancer tissues (*P* = .032) (Table 2). Kaplan‐Meier indicated that patients expressed high MSI2 or low ISYNA1 had a worse survival time (*P* = .005 and *P* = .015, respectively) (Figure 2B,C). Moreover, patients expressed high MSI2 and low ISYNA1 underwent a much worse prognosis (*P* = .001) (Figure 2D). In addition, univariate analysis showed lymph nodes metastasis and UICC stage were also related to the prognosis of pancreatic cancer patients. And in multivariate analysis, the expression of MSI2 and ISYNA1 were independent unfavourable prognostic indicators for pancreatic cancer (Table 3).

**Table 1:** Correlation of ISYNA1 with clinical parameters in PC patients

Parameters	ISYNA1 expression		*P* value
	Low	High	
No. of patients	55	36	
Age (y)			
≤65	47	28	.404
>65	8	8	
Gender			
Male	36	19	.275
Female	19	17	
Tumour location			
Bead	37	28	.346
Body and tail	18	8	
Tumour size (cm)			
≤2.5	10	13	.083
>2.5	45	23	
Differentiation			
Well	16	10	.431
Moderate	38	23	
Poor	1	3	
T stage			
T1	4	8	.035
T2	43	27	
T3	8	1	
Lymph nodes metastasis			
Negative	44	27	.611
Positive	11	9	
UICC staging			
IA + IIA	42	29	.797
IIB + III + IV	13	7	
Postoperative liver metastasis			
Negative	34	29	.067
Positive	21	29	
Vascular permeation			
Negative	26	26	.030
Positive	29	10	
Perineural permeation			
Negative	46	30	1.000
Positive	9	6	

**Table 2:** Association analysis between MSI2 and ISYNA1 in PC

Parameters		MSI2 expression		*r*	*P*
		Low	High		
	Low	14	41		
ISYNA1 expression				−.225	.032
	High	17	19		

**Table 3:** Univariate and multivariate analysis of clinicopathological factors for survival

Parameters	Median survival (mo)	Univariate analysis *P* (Log‐Rank)	Multivariate analysis Hazard ratio (95% CI）	*P*
Age (≤65/>65 y)	15.8/26.3	.372	–	
Gender (male/female)	15.6/20.5	.791	–	
Tumour location (head/body‐tail)	21.5/10.6	.071	–	
Tumour size (≤2.5/>2.5 cm)	26.3/15.6	.291	–	
Differentiation (well/moderate/poor)	20.697/20.5/5.8	.624	–	
T stage (T1/T2/T3)	26.3/20.697/10.6	.090	–	
Lymph nodes metastasis (negative/positive)	21.5/12.667	.012	2.034 (0.403‐10.263)	.390
UICC staging (IA + IIA/IIB + III + IV)	22.0/17.71	.005	1.207 (0.236‐6.160)	.821
Vascular permeation (negative/positive)	23.6/14.0	.103	–	
Perineural permeation (negative/positive)	20.5/26.3	.906	–	
MSI2 expression (low/high)	26.667/12.667	.005	2.213 (1.102‐4.441)	.025
ISYNA1 expression (low/high)	12.167/26.3	.015	0.472 (0.237‐0.941)	.033

### 3.3 ISYNA1 was downregulated by wtp53 in Capan‐2 and SW1990 cells

**Figure 3:**
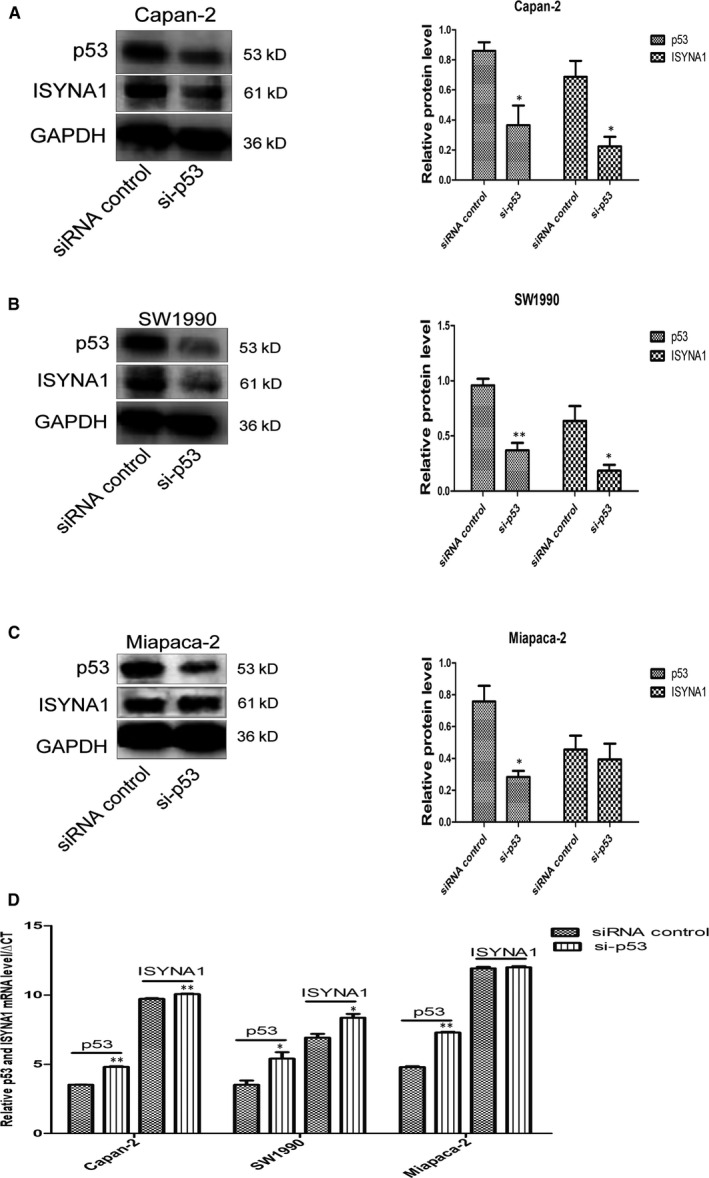
Relationship between p53 and ISYNA1 expression in PC cell lines. WB and qRT‐PCR indicated p53 silencing resulted in decrease of ISYNA1 in wtp53 Capan‐2 and SW1990 cells (A, B and D), but no change of ISYNA1 in mtp53 Miapaca‐2 cell (C and D). Bars indicate Means ± SE,*P < .05, **P < .01

Capan‐2 and SW1990 cells expressed wtp53 while Miapaca‐2 cell expressed mutant p53. The relationship between p53 and ISYNA1 in PC cells remained unclear. WB showed p53 silence decreased ISYNA1 expression in wtp53 Capan‐2 and SW1990 cells (Figure 3A,B), but not in mtp53 Miapaca‐2 cell (Figure 3C). And similar outcomes were also obtained in PCR assays (Figure 3D). It indicated that wtp53 but not mtp53 had a positive regulation targets ISYNA1.

### 3.4 ISYNA1 silencing promoted cell cycle by repressing p21 in Capan‐2 and SW1990 cells

**Figure 4:**
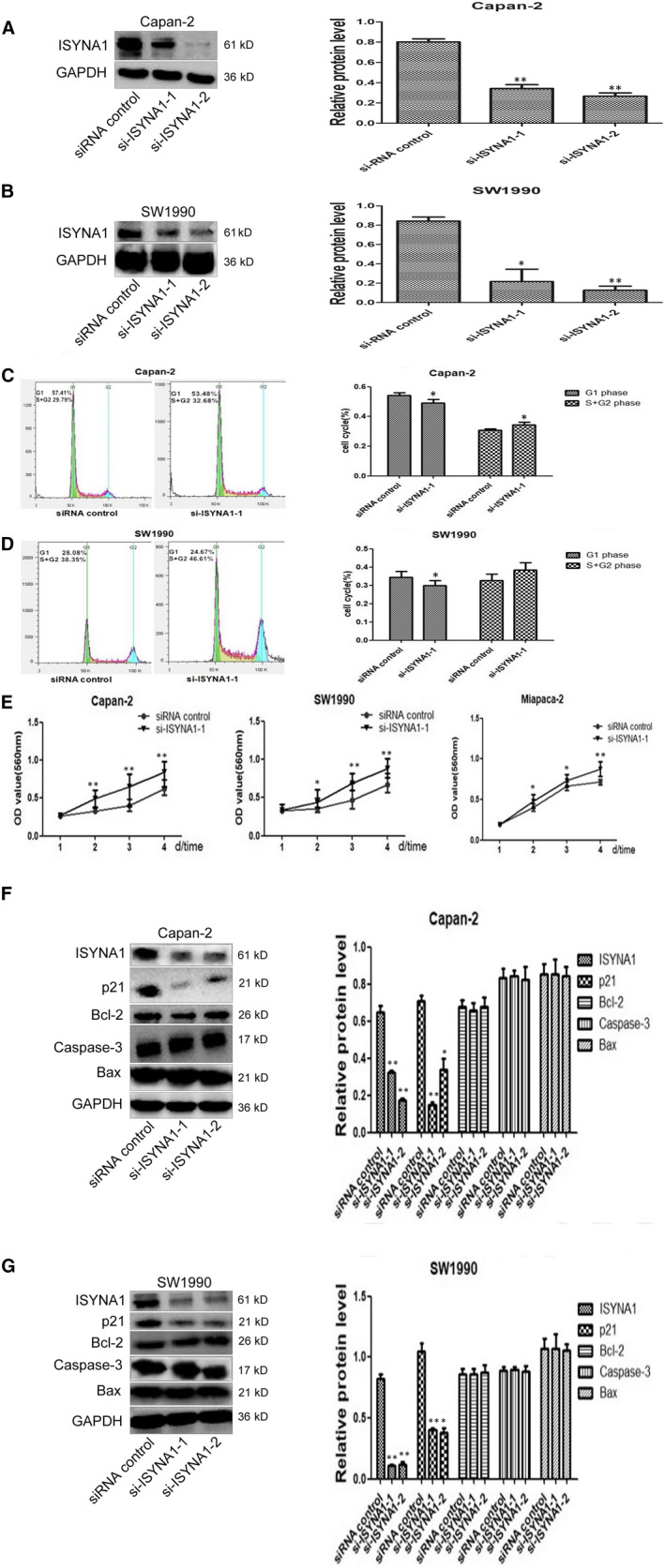
Effects of ISYNA1 silencing on cell cycle in Capan‐2 and SW1990 cells. (A and B) Verification of transfection efficiency by ISYNA1 siRNA sequences in Capan‐2 and SW1990 cells. (C and D) Effects of ISYNA1 silencing on G1 phase and S + G2 phase in Capan‐2 and SW1990 cells, respectively. E, Effects of ISYNA1 silencing on cell proliferation in Capna‐2,SW1990 and Miapaca‐2 cells. F and G, Knocking down of ISYNA1 downregulating p21 in Capan‐2 and SW1990 cells, respectively. Bars indicate Means ± SE,*P < .05, **P < .01

ISYNA1 expression was significantly decreased in ISYNA1 silencing Capan‐2 and SW1990 cells (Figure 4A,B). Flow cytometry showed an obvious decrease of the cell proportion in G1 phase in si‐ISYNA1 group in comparison with siRNA control group in both Capan‐2 (*P* = .016) and SW1990 cells (*P* = .018), while the cell proportion of S + G2 phase was apparently increased in both two cell lines (Figure 4C,D). MTT assay showed cell proliferation in si‐ISYNA1‐1 group was significantly enhanced in Capan‐2, SW1990 and Miapaca‐2 cells (Figure 4E). WB showed p21 protein was significantly downregulated in si‐ISYNA1 groups in both Capan‐2 and SW1990 cells. But other apoptosis‐related proteins (Bax/Bcl2 and caspase‐3) had no obvious change (Figure 4F,G). ISYNA1 silencing promoted cell proliferation and cycle via inhibiting p21 in vitro.

### 3.5 ISYNA1 silencing promoted cell migration and invasion by upregulating ZEB‐1 in Capan‐2 and SW1990 cells

**Figure 5:**
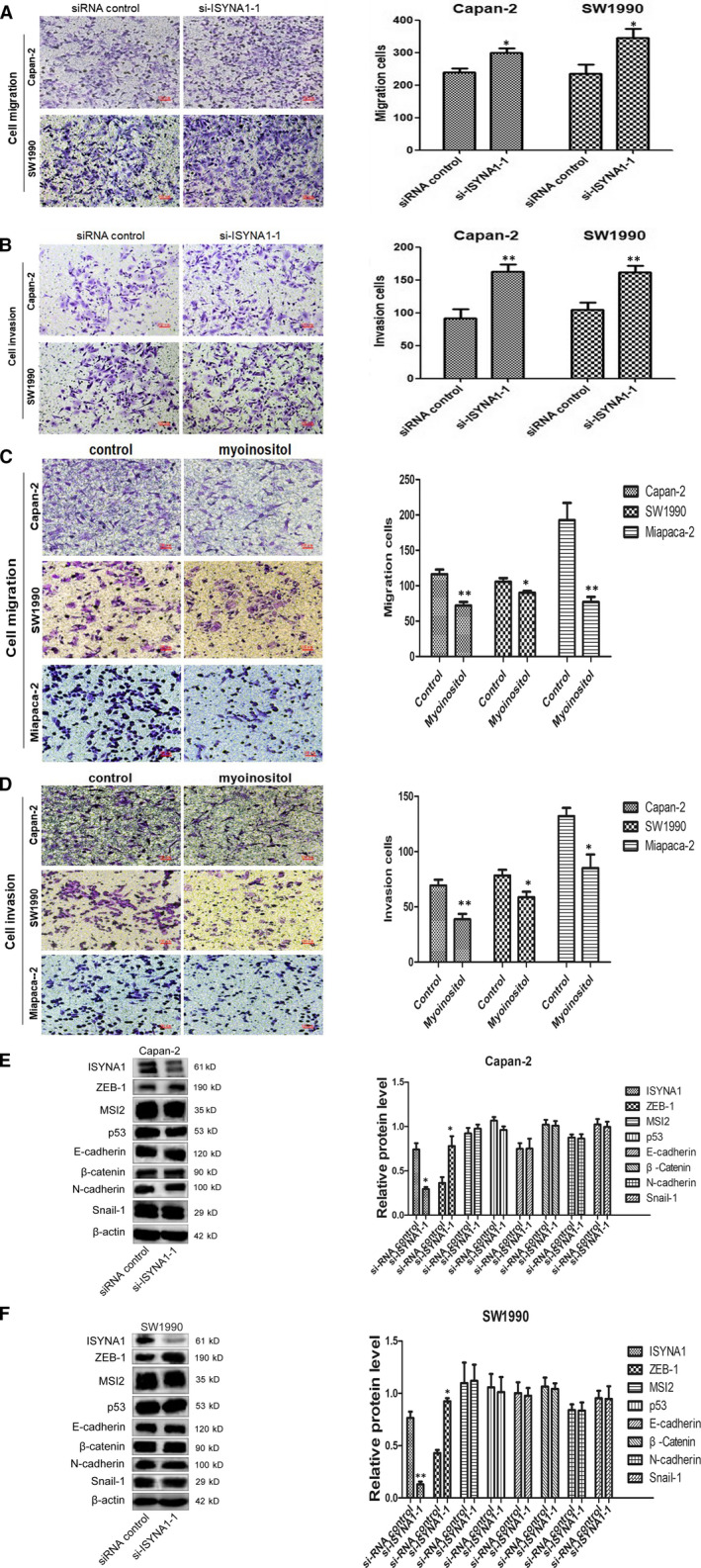
ISYNA1 silencing promoted cell migration and invasion in Capan‐2 and SW1990 cells. A and B, Knocking down of ISYNA1 promoted cell migration and invasion in Capan‐2 and SW1990 cells, respectively (100×). C and D, Addition of myoinositol inhibited cell migration and invasion in Capan‐2 and SW1990 cells, respectively (100×). E and F, WB analysis showed ZEB‐1 was upregulated in ISYNA1 knocked down PC cells. Bars indicate Means ± SE,*P < .05, **P < .01

Transwell assay demonstrated that cell migration and invasion was prominently increased in si‐ISYNA1 group in comparison to siRNA control group in Capan‐2 and SW1990 cells (Figure 5A,B). Myoinositol, as a kind of water‐soluble vitamin synthesized, acts as a tumour suppressive role in various cancers. However, its function remains unclear in PC. We found that myoinositol significantly suppressed cell migration and invasion in vitro (Figure 5C,D). WB showed an evident upregulation of ZEB‐1 in si‐ISYNA1 groups compared with control, but other proteins (MSI2, p53, E‐cadherin, β‐Catenin, N‐cadherin and snail‐1) were unchanged (Figure 5E,F). Altogether, ISYNA1 silence promoted cell migration and invasion by upregulating ZEB‐1 in Capan‐2 and SW1990 cells.

### 3.6 ISYNA1 silencing reversed reduction of cell migration and invasion caused by MSI2 silence in Capan‐2 and SW1990 cells

**Figure 6:**
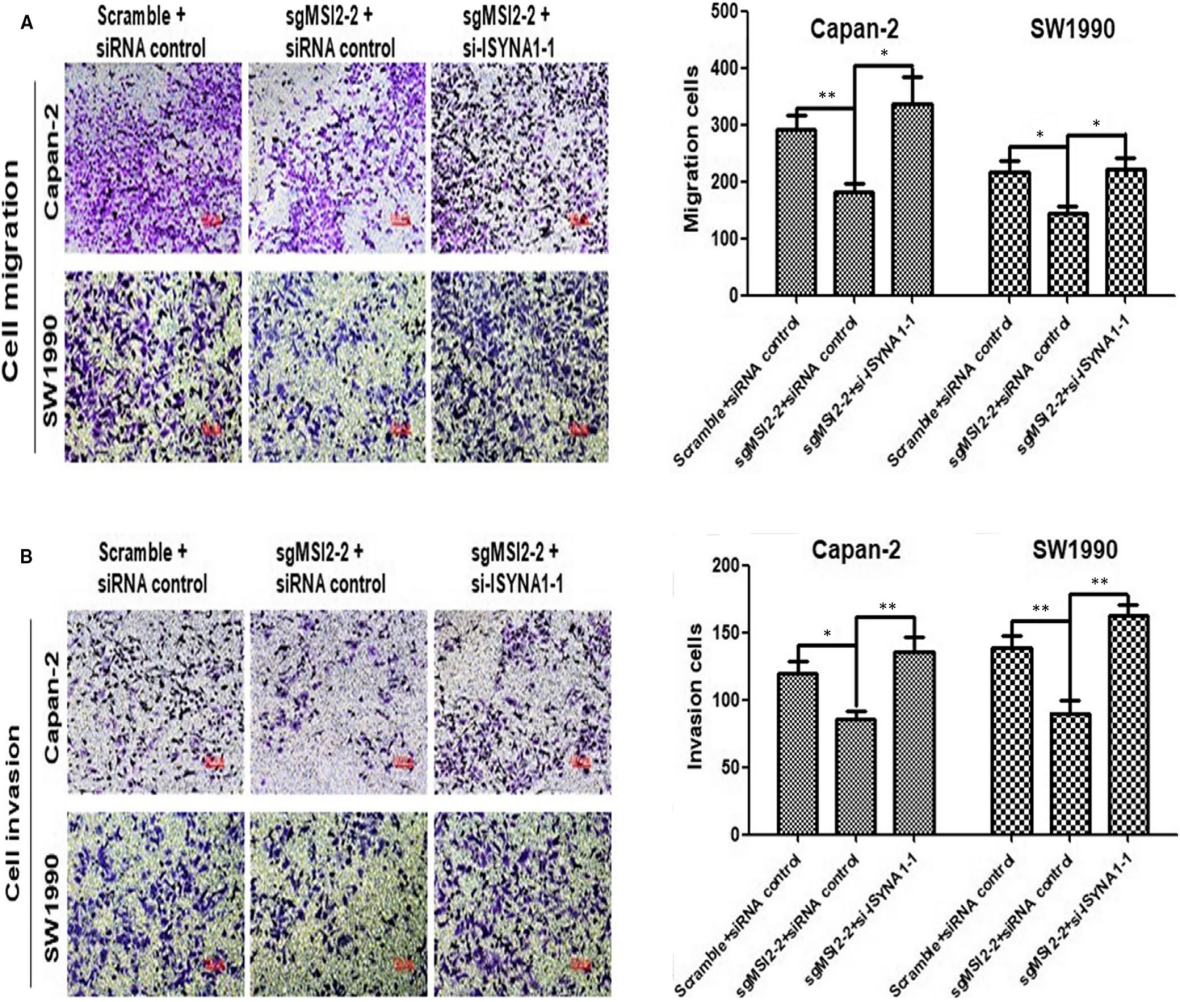
A and B, Knocking down of ISYNA1 reversed effects of cell migration and invasion induced by MSI2 silencing in Capan‐2 and SW1990 cells, respectively (×100). Bars indicate Means ± S.E,*P < .05, **P < .01

**Figure 7:**
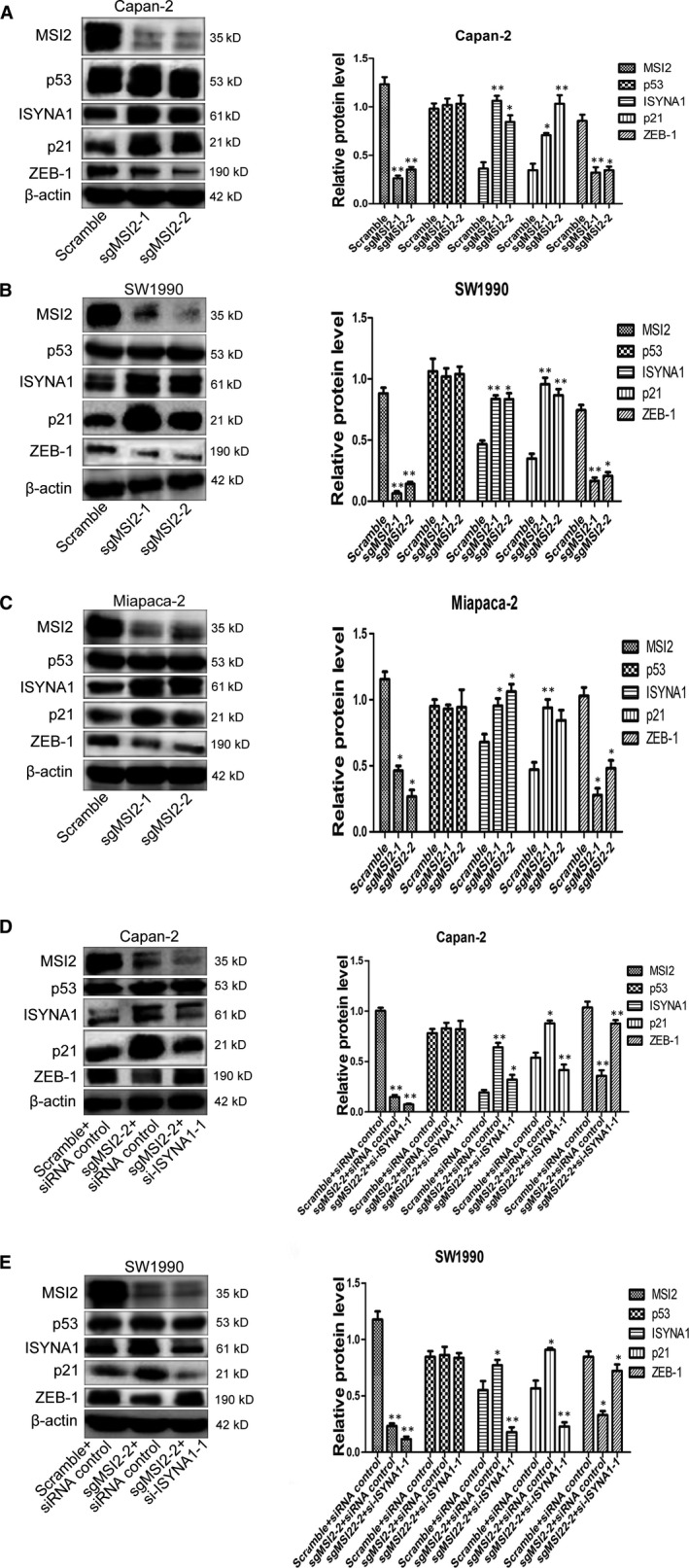
Relationship of MSI2, p53, ISYNA1, p21 and ZEB‐1 in Capan‐2 and SW1990 cells by WB analysis. A‐C, Effects of MSI2 silencing on p53, ISYNA1, p21 and ZEB‐1 in Capan‐2,SW1990 and Miapaca‐2 cells. D and E, ISYNA1 silencing reversed change of p21 and ZEB‐1 in MSI2‐silencing Capan‐2 and SW1990 cells. Bars indicate Means ± S.E,*P < .05, **P < .01

Crispr‐cas9 mediated MSI2 silencing Capan‐2 and SW1990 stable cells were successfully constructed (Figure 7A,B), which were co‐transfected with ISYNA1siRNA. Transwell assays indicated cell migration was prominently decreased in MSI2 silencing Capan‐2 and SW1990 cells compared with control group. However, ISYNA1 silence obviously reversed reduction of cell migration caused by MSI2 silence (Figure 6A). Similarly, ISYNA1 silence reversed the reduction of cell invasion resulted from MSI2 silence in Capan‐2 and SW1990 cells (Figure 6B). Taken together, MSI2 promoted cell migration and invasion by downregulating ISYNA1 protein in Capan‐2 and SW1990 cells.

### 3.7 MSI2 and ISYNA1 coordinately regulated p21 and ZEB‐1 in vitro

We next researched the potential relationship of MSI2, p53, ISYNA1, p21 and ZEB‐1 in vitro. WB showed MSI2 silence upregulated ISYNA1 and p21 but downregulated ZEB‐1 in Capan‐2, SW1990 and Miapaca‐2 cells, while p53 expression (both wtp53 and mtp53) was unchanged (Figure 7A‐C). However, ISYNA1 silence significantly reversed the upregulation of ISYNA1 and p21, and the downregulation of ZEB‐1 induced by MSI2 silence in the Capan‐2 and SW1990 cells (Figure 7D,E). Our previous study also showed no regulation of MSI2 towards wtp53 and mtp53 in normal PC cells.^9^ Taken together, MSI2 downregulated p21 and upregulated ZEB‐1 protein via inhibiting ISYNA1 protein in p53‐independent manner.

## 4 DISCUSSION

Our previous studies indicated that MSI2 overexpression made for the progression of pancreatic cancer by negative regulation of Numb and wtp53 with the stimulation of gemcitabine or cisplatin.^8^, ^9^ In current study, we further investigated the novel signalling in terms of MSI2 in PC tissue and cell level. We identified ISYNA1 as a novel candidate tumour suppressor regulated by MSI2 and wtp53, which has to been reported, to our knowledge.

ISYNA1 encodes an inositol‐3‐phosphate synthase enzyme and plays a vital role in cellular myoinositol biosynthesis, which is closely related to the tumorigenesis.^14^ In current study, ISYNA1 was downregulated in pancreatic cancer tissues and was negatively related to MSI2 expression, T stage, vascular permeation and poor prognosis of PC patients. Meanwhile, patients expressed high MSI2 and low ISYNA1 underwent a worse prognosis all which is not reported previously to our knowledge. Previous studies focus on ISYNA1 function in gene differential methylation and isoforms identification.^19^, ^20^ Recently, a high concentration of myoinositol in tumours is found related to high levels of ISYNA1.^21^ However, studies involved the role of ISYNA1 in malignant tumours remain scarce and controvertial. The expression of ISYNA1 and myoinositol in gliomas was significantly higher than that in primary central nervous system lymphomas.^22^ Overexpression of ISYNA1 suppressed cell proliferation in HCT116 and HepG2 cells.^15^ The inconsistent outcomes might be due to the difference in cancer types and environment.

Our previous study reported that MSI2 silencing upregulated wtp53 only under chemotherapy stimulus.^9^ Here, we first found that MSI2 silencing upregulated ISYNA1 and identified ISYNA1 as a direct target of wtp53 in Capan‐2 and SW1990 cells. However, in mtp53 Miapaca‐2 cell, p53 silence had no effects on ISYNA1. Also, MSI2 silence had no effect in both wtp53 and mtp53 in normal PC cells in our current and previous studies.^9^ Thus, ISYNA is a downstream target of MSI2 and wtp53 via different signal pathways in the progression of PC.

ISYNA1 silence promoted cell proliferation and cycle through downregulating p21 protein which is a classic marker in terms of cell growth and cycle in PC by regulating G1 and S phase.^23^, ^24^ Previous studies showed that p53 interacted with p21 and formed a p53/p21 complex that regulated cell invasion and apoptosis by targeting Bcl‐2 protein ^25^; p53 simultaneously controls multiple pathways to induce cellular senescence through p21 and Akt.^26^In present study, ISYNA1, as a direct target of p53, regulated cell proliferation and cycle by inhibiting p21 in PC cells which indicated that p53, ISYNA1 and p21 play a crucial role in the development and progression of pancreatic cancer. Moreover, MSI2 silence upregulated ISYNA1 and p21 proteins in PC cells which was reversed by ISYNA1 silence. Thus, overexpression of MSI2 promoted cell cycle of PC cells by downregulating ISYNA1‐p21 pathway.

ZEB‐1, a member of ZHF family, was closely associated with epithelial‐mesenchymal transition in many tumours, such as colorectal cancer and PC.^27^, ^28^, ^29^ Its abnormal expression contributed to the invasion and metastasis of carcinoma by inducing EMT.^30^ Here, we firstly reported that ISYNA1 silence promoted cell migration and invasion via upregulating ZEB‐1 in PC. Furthermore, ISYNA1 silence reversed the reduction of cell migration, cell invasion and ZEB1 protein resulted from MSI2 silence in vitro. Thus, the overexpression of MSI2 facilitates cell migration and cell invasion in pancreatic cancer by regulating ISYNA1‐ZEB‐1 pathway.

In summary, MSI2 accelerates the development and progression of pancreatic cancer through a novel ISYNA1‐p21/ZEB‐1 pathway, which supplies a novel gene targets in the PC treatment.

## CONFLICT OF INTEREST

No conflicts of interest are confirmed.

## AUTHOR CONTRIBUTION

**Lei Zhou:** Conceptualization (equal); Data curation (lead); Formal analysis (lead); Investigation (equal); Methodology (equal); Software (equal); Writing‐original draft (lead); Writing‐review & editing (lead). **WeiWei Sheng:** Conceptualization (lead); Data curation (lead); Formal analysis (lead); Methodology (equal); Validation (equal); Visualization (equal); Writing‐original draft (equal); Writing‐review & editing (equal). **Chao Jia:** Data curation (equal); Formal analysis (equal); Methodology (equal); Visualization (equal). **Xiaoyang Shi:** Investigation (equal); Methodology (equal); Software (equal). **Rongxian Cao:** Data curation (equal); Methodology (equal). **Guosen Wang:** Formal analysis (supporting); Methodology (supporting). **Yiheng Lin:** Formal analysis (supporting); Methodology (supporting). **Fang Zhu:** Supervision (supporting); Validation (supporting). **Qi Dong:** Supervision (supporting); Validation (supporting). **Ming Dong:** Conceptualization (equal); Data curation (equal); Formal analysis (equal); Funding acquisition (equal); Investigation (equal); Project administration (equal); Resources (equal); Supervision (equal); Validation (equal); Writing‐original draft (equal); Writing‐review & editing (equal).

## Supporting information

Table S1

## Data Availability

All data will be available upon reasonable request.
