# Supplementary material for: Musashi2 promotes the progression of pancreatic cancer through a novel ISYNA1‐p21/ZEB‐1 pathway
Source: J Cell Mol Med. 2020 Aug 11;24(18):10560–72. doi: 10.1111/jcmm.15676 (PMC7521282; doi:10.1111/jcmm.15676)
Supplement: Supplementary file 1 — Table S1 [file JCMM-24-10560-s001.doc]

**Supplementary Table Primer sequences and target gene sequences for interference**

| Oligo Name |  | Primer/target sequences | |
| --- | --- | --- | --- |
| ISYNA1 | sense（5'-3'） TCCGCTCTAAGGAGGTGTCC | | |
|  | antisense（5'-3'） CGGCACATACTTGATGACCAC | | |
| GAPDH | sense（5'-3'） CATGAGAAGTATGACAACAGCCT | | |
|  | antisense（5'-3'） AGTCCTCCACGATACCAAAGT | | |
| Scramble  (sgRNA control) | CGCTTCCGCGGCCCGTTCAA | | |
| sgMSI2-1 | TCCTCGTCGAGCGCAACCCA | | |
| sgMSI2-2 | CCATGAGTTAGATTCCAAGA | | |
| siRNA control | sense（5'-3'） | | UUCUCCGAACGUGUCACGUTT |
|  | antisense（5'-3'） | | UUCUCCGAACGUGUCACGUTT |
| si-ISYNA1-1 | sense（5'-3'） | | GGAGAUGACUUCAAGUCAGTT |
|  | antisense（5'-3'） | | CUGACUUGAAGUCAUCUCCTT |
| si-ISYNA1-2 | sense（5'-3'） | | CCACCUACCCUAUGUUGAATT |
|  | antisense（5'-3'） | | UUCAACAUAGGGUAGGUGGTT |
| si-p53 | sense（5'-3'） | | CUACUUCCUGAAAACAACGTT |
|  | antisense（5'-3'） | | CGUUGUUUUCAGGAAGUAGTT |
